# PZT-Based Ultrasonic Guided Wave Frequency Dispersion Characteristics of Tubular Structures for Different Interfacial Boundaries

**DOI:** 10.3390/s18124111

**Published:** 2018-11-23

**Authors:** Shi Yan, Bowen Zhang, Gangbing Song, Jiaoyun Lin

**Affiliations:** 1 School of Civil Engineering, Shenyang Jianzhu University, Shenyang 110168, China; jxx0829@stu.sjzu.edu.cn; 2 Department of Mechanical Engineering, University of Houston, Houston, TX 77204, USA; gsong@uh.edu

**Keywords:** tubular structures, longitudinal ultrasonic guided waves (UGWs), dispersion characteristics, interfacial boundary conditions, product of frequency and thickness, interfacial debonding damage, PZT-based transducers

## Abstract

For tubular structures, ultrasonic guided waves (UGWs) which are closely related to interfacial boundary conditions such as gas, liquid and solid materials, are usually used in damage detection. Due to the different phase materials inside tubes, the interfacial boundary (connection) conditions are variable, which has a great influence on the dispersion-related UGW propagation characteristics. However, most UGW-based damage detection methods only consider the pipeline structures as hollow tubes, ignoring the interfacial boundary condition influences on the UGW propagation. Based on the UGW theory, this paper aims to propose a novel method for describing the UGW propagation characteristics for different interfaces, and lay a foundation for the UGW-based tubular structure damage detection. Based on the Navier’s equation of motion and combined with interfacial boundary conditions and coordinate conditions, the dispersion equations for a hollow steel tube, a tube filled with liquid, and a concrete filled steel tube (CFST) were established, respectively. Under the given conditions of both materials and geometric parameters, the transcendental dispersion equations were established and solved by using a numerical method. The UGW propagation characteristics in different interfaces were classified and discussed, and the dispersion curves of both group and phase velocities are drawn. To validate the efficiency of theoretical and numerical results, three kinds of model tubular structure experiments filled in air (hollow), water and concrete, respectively, were performed based on lead zirconate titanate (PZT) transducer UGWs. The results showed that the UGWs propagation in different interfaces has the dispersion and multi-modes characters, which are not only related to the product of frequency and thickness, but also to the internal dielectric material parameters and interfacial boundary conditions.

## 1. Introduction

Tubular structures are widely used in bridges, underground pipes, pipelines and high-rise buildings, etc. Due to environmental impacts, material aging effects and overloading, these tubular structures may experience various forms of interfacial damage during the construction and service phase, and their whole lifespan reliability might be reduced to a certain extent. Therefore, it is necessary to monitor or identify the interfacial defects such as debonding in tubular structures by using non-destructive testing (NDT) methods [1,2,3,4].

The existing damage detection (DD) and structural health monitoring (SHM) methods are usually classified into artificial percussion methods, ultrasound-based methods, optical fiber-based methods and so on. However, these technologies usually need to be used for examining the monitored tubular structure point by point, which is time-consuming, laborious and cumbersome during the detection process. The ultrasonic guided wave (UGW)-based detection technology can overcome those disadvantages with its many advantages, such as high efficiency, high speed and wide range of detection, and has been widely used in structural defect detection [5]. As the wave is traveling back and forth between the interfaces that make up the waveguide, the round-trip wave produces a complex waveform and interferences. If the guided wave propagates over an infinite plate or a pipe at two parallel interfaces, it will travel along the plate or pipe surface. Therefore, as the guided wave propagates through a cylindrical shell, a rod and a layered elastic body, it will generally propagate along their axial directions. Therefore, flat plates and cylindrical shells, rods and laminated elastic bodies are typical waveguides for guided wave propagations [6,7]. Because of the multi-modes and frequency dispersion characteristics of an UGW, it becomes very complicated during excitation, propagation and reception. In order to make a better use of UGWs to detect damages in structures, a proper mode and frequency of the guided wave should be firstly selected and applied in DDs and SHMs. The dispersion curves which show the relationship between group or phase velocities and frequencies for UGWs have become an important reference, and this becomes a prerequisite for the use of UGWs in a test or engineering. Especially, as the time of flight (ToF) method is used for defects localization, the frequency dispersive characteristics of the selected UGW is quite dominated for evaluating the guided wave group (or phase) velocity. Based on the UGW theory, this paper aims to propose a novel method for describing the UGW propagation characteristics in different interfaces, and lay a foundation for the UGW-based tubular structure damage detection or health monitoring.

Research into guided waves and their applications in engineering has a long history and has produced plenty of fruitful research. The objective of an investigation on guided waves can be classified into four stages, which are the stages of plate structures with free boundary, hollow cylindrical shells, multi-layer composite pipelines, and composite structures such as concrete filled steel tubes (CFST). The research results have covered from simple issues to complicated ones.

Rayleigh [8] and Lamb [9] studied the propagation properties of the elastic waves in a free state in an isotropic elastic plate, and obtained the transcendental equation of the monolayer isotropic elastic plate, named the Rayleigh-Lamb transcendental equation. Later, Lamb obtained the wave equation under the free boundary condition of the plate, and obtained a special set of wave solutions, which promoted the development of guided wave theory.

For hollow cylindrical shells, Ghosh [10] first carried on the linear solution derivation of guided wave transmission. Then, Love [11] described a stress wave propagation analysis. Cooper and Naghdi [12] and Naghdi and Cooper [13] used the same theory to further study and analyze the propagation law of non-axisymmetric waves in a hollow cylindrical shell, respectively, but never obtained a comprehensive numerical solution for the guided wave propagation in the cylindrical shell structure. Finally, Gazis [14] on the basis of the existing results, analyzed the imperfections of the plate and shell theory, and used the linear elasticity theory to solve the infinite long isotropic cylindrical shell problem. Gazis [15,16] also obtained the dispersion equation of the longitudinal mode and the torsion mode, and the dispersion curve of the multi-mode was drawn by a numerical calculation, and the cutoff frequency of each mode was obtained according to the dispersion curve. 

For a multilayer structure system, the research methods are classified as the transfer matrix method, global matrix method, analytic method, numerical analysis method and semi-analytical finite element method, etc. Lowe [17,18] used the transfer matrix method to establish the dispersion equation of Lamb wave in a multilayer plate, then, he deduced the dispersion equation of the guided wave in the layered cylinder. Markus et al. [19], and Yuan and Hsieh [20] used the analytical method to study the propagation characteristics of the wave in the free composite cylindrical shell. Xi et al. [21] studied the wave propagation characteristics in evacuated composite cylindrical shells and liquid filled composite cylindrical shells by a semi-analytical method. Huang et al. [22] studied the dispersion characteristics of waves in composite cylindrical shells by a numerical dispersion method. Based on axisymmetric guided waves, Du et al. [23] analyzed the differences between the interface changes, and explored the dispersion equations of cylindrical composite structures, and obtained the dispersion equation of a weakly confined interface double layer cylindrical structure in the interface spring model. In addition, experiments were carried out on the double layered composite cylindrical structure. The double layered composite rods are filled with liquid with the inner layer free interface and zero inner radius, obtaining the phase velocity which is an effective factor of interface characteristics of low order guided waves.

The mechanism for guided wave propagation in multilayer composite cylindrical structures can be extended into composite structures. For example, Xu et al. [24] showed that guided waves can be applied in concrete filled steel tubular structures to detect damages such as interfacial debonding between concrete and steel pipe, and the filled concrete can be considered as a boundary of the steel pipe. 

So far, the propagation characteristics of UGWs in tubular structures filled with air (hollow) or liquid have been extensively studied and applied in damage detection. The current challenging issue is that the characteristics of UWGs propagating in different interfaces have not been systematically studied and applied in engineering. A systematic and effective method for detecting interface damage using UWGs is actually required. However, one of challenges for DDs and SHMs using UGWs is the frequency dispersion influence, especially considering boundary conditions, on the UGW propagation which makes it much complicated and reduces the precision of DD and SHM evaluations. Therefore, this paper takes the tubular structures as the research objective, and numerical analysis is used to solve the dispersion equation of the guided wave in the tubular structures with different interfacial boundary conditions. Then, the MATLAB software is applied to analyze and draw the group velocity and phase velocity dispersion curves. The developed frequency dispersion curves are finally validated by an experiment. This paper also simulates the interface debonding by setting the artificial damage, the appropriate actuation frequency is selected according to the dispersion curve, and the damage is identified by the signal energy method. The feasibility of using the energy method to detect the interface damage is verified, which can lay a foundation for the detection of interface damage of tubular structures by using UGWs.

## 2. Dispersion Equations for Tubular Structures and Solution

### 2.1. Dispersion Equations for Tubular Structures

#### 2.1.1. For Hollow Tubular Structures

**Figure 1:**
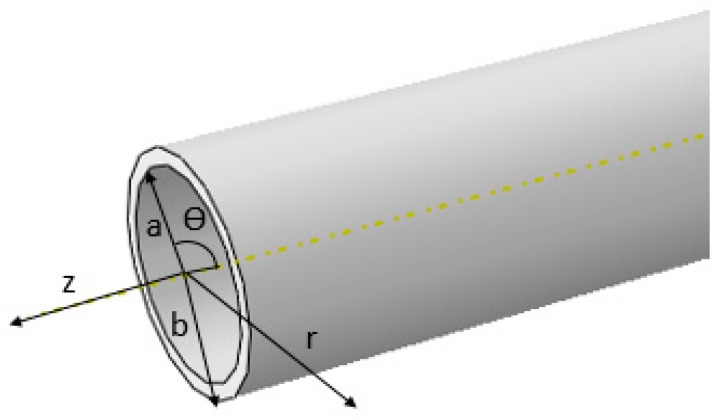
The schematic of a cylindrical guided wave in a tubular structure.

Figure 1 is the tubular structural model. It is assumed that the z direction is infinite, according to the elastic mechanics theory, when the guided waves are propagating in the structure, the displacements of the particles must satisfy the Navier wave equations of motion [14,25], as shown in Equation (1). According to the wave equation, the displacement and stress expressions of the guided wave in the inner material and the outer steel tube are established, respectively. The displacement and stress relations of the adjacent layers are established by using boundary conditions and interfacial coordinate conditions. Meanwhile, the dispersion equation in tubular structures with different boundary conditions is obtained by the above equations:

(1) $$\mu \nabla^{2}U+(\lambda +\mu )\nabla (\nabla \cdot U)=\rho \frac{\partial^{2}U}{\partial t^{2}}$$

in which, *U* is displacement; *t* is travel time; *μ* and *λ* is Lame constants of materials; *ρ* is density of material.∇ is Laplace operator.

When there is no external force, the displacement vector *U* can be decomposed into an expression of an expansion scalar potential function φ and an equal volume vector potential function *H* according to the Helmholtz decomposition law, as shown in Equation (2):

(2) U=∇φ+∇×H

in which, ∇⋅H=0.

For Equation (2), the potential function ϕ and *H* should be satisfied:

(3) $$c_{L}{}^{2}\nabla^{2}\varphi =\frac{\partial^{2}\varphi }{\partial t^{2}}$$

(4) $$c_{T}{}^{2}\nabla^{2}H=\frac{\partial^{2}H}{\partial t^{2}}$$

in which, $c_{L}$ and $c_{T}$ are P-wave and S-wave velocity in elastic medium, respectively, as shown in Equation (5):

(5) $$c_{L}=\sqrt{(\lambda +2\mu )/\rho },c_{T}=\sqrt{\mu /\rho }$$

For the propagation of guided waves in a pipe structure, Gazis [14] first studied and gave the exact solution of this boundary condition:

(6) $$\left\{ \begin{array}{l} \varphi =f(r)\cos n\theta \cos(\omega t+kz)\\ H_{r}=g_{r}(r)\sin n\theta \sin(\omega t+kz)\\ H_{\theta }=g_{\theta }(r)\cos n\theta \sin(\omega t+kz)\\ H_{z}=g_{z}(r)\sin n\theta \cos(\omega t+kz) \end{array}\right.$$

in which, subscripts *r, θ* and z are radial, circumferential and axial, respectively. Substituting Equation (6) into Equations (3) and (4):

(7) $$\left\{ \begin{array}{l} (\nabla^{2}+\frac{\omega^{2}}{c_{L}{}^{2}})\varphi =0\\ (\nabla^{2}+\frac{\omega^{2}}{c_{T}{}^{2}})H_{z}=0\\ (\nabla^{2}-\frac{1}{r^{2}}+\frac{\omega^{2}}{c_{L}{}^{2}})H_{\theta }+\frac{2}{r^{2}}\frac{\partial H_{\theta }}{\partial \theta }=0\\ (\nabla^{2}-\frac{1}{r^{2}}+\frac{\omega^{2}}{c_{T}{}^{2}})H_{r}-\frac{2}{r^{2}}\frac{\partial H_{r}}{\partial \theta }=0 \end{array}\right.$$

Introducing differential operators:

$$B_{n,x}=\left[\frac{\partial^{2}}{\partial x^{2}}+\frac{1}{x}\cdot \frac{\partial }{\partial x}-\left(\frac{n^{2}}{x^{2}}-1\right)\right]$$

Obtaining Equation (8):

(8) $$\left\{ \begin{array}{l} B_{n,\alpha r}[f]=0\\ B_{n,\beta r}[g_{3}]=0\\ B_{n+1,\beta r}[g_{r}-g_{\theta }]=0\\ B_{n-1,\beta r}[g_{r}+g_{\theta }]=0 \end{array}\right.$$

in which:

(9) $$\alpha^{{\left(m\right)}^{2}}=\frac{\omega^{2}}{c_{L}^{{\left(m\right)}^{2}}}-k^{2},\beta^{{\left(m\right)}^{2}}=\frac{\omega^{2}}{c_{T}^{{\left(m\right)}^{2}}}-k^{2}$$

The general solution of Equation (8) can be found by the Bessel function:

(10) $$\left\{ \begin{array}{l} f_{m}=A_{m}\cdot Z_{n}(\alpha^{m}\cdot r)+B_{m}W_{n}(\alpha^{m}\cdot r)\\ g_{m}=C_{m}\cdot Z_{n+1}(\beta^{m}\cdot r)+D_{m}W_{n+1}(\beta^{m}\cdot r)\\ g_{m+2}=E_{m}\cdot Z_{n}(\beta^{m}\cdot r)+F_{m}W_{n}(\beta^{m}\cdot r) \end{array}\right.$$

in which, *Z*_n_ represents Bessel functions J and Y; *W*_n_ denotes modified Bessel functions *I* and *K*; *m* is the number of pipeline structure layers.

When $g_{2}=0$:

(11) $$g_{r}=g_{\theta }=g_{1}$$

The solution of the displacement field is:

(12) {urm=[f′+(nr)gm+2+kgm]cosnθcos(ωt+kz)uθm=[−(nr)fm+kgm−gm+2′]sinnθcos(ωt+kz)uzm=[−kfm+(n+1)(gmr)−gm+2′]cosnθsin(ωt+kz)

in which, $\mu_{r}$, $\mu_{\theta }$ and $\mu_{z}$ are radial component, circumferential component and axial component in displacement field, respectively. According to the knowledge of elastic mechanics, the strain-displacement relation can be obtained, as shown in Equation (13):

(13) $$\left\{ \begin{array}{l} \varepsilon_{rr}^{m}=\partial u_{r}^{m}/\partial r\\ \varepsilon_{rz}^{m}=\left(1/2\right)\left(\partial u_{r}^{m}/\partial z+\partial u_{z}^{m}/\partial r\right)\\ \varepsilon_{r\theta }^{m}=\left(1/2\right)\left[r\frac{\partial }{\partial r}\left(\frac{u_{\theta }^{m}}{r}\right)+\frac{1}{r}\frac{\partial u_{r}^{m}}{\partial \theta }\right] \end{array}\right.$$

The stress-strain relationship is:

(14) $$\left\{ \begin{array}{l} \sigma_{rr}^{m}=\lambda_{m}\Delta +2\mu_{m}\varepsilon_{rr}^{m}\\ \sigma_{rz}^{m}=2\mu_{m}\varepsilon_{rz}^{m}\\ \sigma_{r\theta }^{m}=2\mu_{m}\varepsilon_{r\theta }^{m} \end{array}\right.$$

in which, Δ represents the volume expansion ratio:

(15) $$\Delta =\nabla^{2}\varphi =-(\alpha^{\left(m\right)}{}^{2}+k^{2})f\cos n\theta \cos(\omega t+kz)$$

Simultaneous Equations (12)–(15), the stress component of the stress field can then be obtained:

(16) $$\begin{array}{l} \sigma_{rr}^{m}=\{-\lambda_{m}(\alpha^{\left(m\right)}{}^{2}+k^{2})f_{m}+2\mu_{m}[f_{m}{}^{\prime \prime }+\frac{n}{r}(g_{m+2}{}^{\prime }-\frac{g_{m+2}}{r})+kg_{m}{}^{\prime }]\}\cos n\theta \cos(\omega t+kz)\\ \sigma_{r\theta }^{m}=\mu_{m}[-\frac{2n}{r}(f_{m}{}^{\prime }-\frac{f_{m}}{r})-(2g_{m+2}^{\prime \prime }+\beta^{\left(m\right)}{}^{2}g_{m+2})-k(\frac{n+1}{r}g_{m}-g_{m}^{\prime })]\sin n\theta \cos(\omega t+kz)\\ \sigma_{rz}=\mu_{m}\{-2kf_{m}{}^{\prime }-\frac{n}{r}g_{m}^{\prime }+\frac{n}{r}(\frac{n+1}{r}-\beta^{\left(m\right)}{}^{2}+k^{2})g_{m}-\frac{nk}{r}g_{m+2}\}\cos n\theta \sin(\omega t+kz) \end{array}$$

When the inside of the pipe is air, the stress on the inner and outer surfaces should meet the following boundary conditions:

(17) $${\sigma_{rr}|}_{r=a,b}=0,\begin{array}{c} {\sigma_{rz}|}_{r=a,b}=0, \end{array}\begin{array}{c} {\sigma_{r\theta }|}_{r=a,b}=0, \end{array}$$

Substituting Equation (16) into the boundary condition Equation (17), then:

[*c_ij_*]_6×6_[*A B A*_1_*B*_1_*A*_2_*B*_2_]^T^ = [0 0 0 0 0 0]^T^ (18)

In order to make the Equation (18) have a non-zero solution, the coefficient determinant must be zero:

| *c_ij_* | = 0 (*i*, *j* = 1…6) (19)

Equation (19) is the dispersion equation of the UGWs in the hollow tubular.

#### 2.1.2. For Tubular Structures Filled with Liquid

In the tube filled with liquid, the boundary condition at the outer surface of the pipe (r=b) is:

(20) $$\left\{ \begin{array}{l} {\left(\sigma_{rr}\right)}_{r=b}=0\\ {\left(\sigma_{rz}\right)}_{r=b}=0 \end{array}\right.$$

Since the inside of the tube is filled with liquid, the inner surface of the tube is in close contact with the liquid column in the tube, so the radial displacement and radial stress component at the boundary between the inner surface of the tube and the liquid in the tube are continuous. For the liquid in the tube is a non-viscous liquid, the liquid in the tube does not bear the shearing force, so the stress component along the center line of the tube at the boundary between the inner surface of the tube and the liquid in the tube is $\sigma_{rz}=0$. The boundary condition at the inner surface of the tube is:

(21) $$\left\{ \begin{array}{l} {\left(u_{r}\right)}_{r=a}={\left(u_{r}^{f}\right)}_{r=a}\\ {\left(\sigma_{rr}\right)}_{r=a}={\left(\sigma_{rr}^{f}\right)}_{r=a}\\ {\left(\sigma_{rz}\right)}_{r=a}={\left(\sigma_{rz}^{f}\right)}_{r=a}=0 \end{array}\right.$$

When the guided wave propagates in a non-viscous liquid cylinder, the Navier displacement equilibrium equation is still satisfied. However, the displacement field $U^{f}$ can only be represented by a scalar potential ϕ and there is no vector potential H. The displacement field $U^{f}$ is expressed as follows:

(22) $$U^{f}=\nabla \phi^{f}$$

The condition for the Navier displacement equilibrium equation is:

$${\left(c_{L}^{f}\right)}^{2}\nabla^{2}\phi^{f}=\frac{\partial^{2}\phi^{f}}{\partial^{2}t}$$

According to Gazis’ governing equations for the exact solution of this boundary value problem, we assume:

(23) $$\varphi^{f}=f^{f}(r)\cos n\theta \cos(\omega t+kz)$$

Substituting Equation (23) into the Equation (22):

(24) $$(\nabla^{2}+\frac{\omega^{2}}{c_{L}^{f}{}^{2}})\varphi^{f}=0$$

By introducing a differential operator, combined with the Bessel equation and the integral invariant property, the solution of the displacement field can be obtained as:

(25) $$u_{r}^{f}={\left(f^{f}\right)}^{\prime }\cos n\theta \cos(\omega t+kz)$$

According to the relevant theory of elastic mechanics, the relationship between strain and displacement can be expressed as:

(26) $$\varepsilon_{rr}^{f}=(\partial u_{r}^{f}/\partial r)$$

The stress-strain relationship can be expressed as:

(27) $$\sigma_{rr}^{f}=\lambda^{f}\Delta +2\mu^{f}\varepsilon_{rr}^{f}$$

In which, Δ represents the volume expansion ratio:

(28) $$\Delta =\nabla^{2}\varphi^{f}=-[{\left(\alpha^{f}\right)}^{2}+k^{2}]f^{f}\cos n\theta \cos(\omega t+kz)$$

Simultaneous Equations (25)–(28), the stress component of the stress field can be obtained as:

(29) $$\sigma_{rr}^{f}=\{-\lambda^{f}[{\left(\alpha^{f}\right)}^{2}+k^{2}]f_{1}^{f}+2\mu^{f}{\left(f_{1}^{f}\right)}^{\prime \prime }\}\cos(n\theta )\cos(wt+kz)$$

Finally giving Equation (30):

(30) $$u_{r}^{f}=U_{r}^{f}(r)\cos(n\theta )\cos(wt+kz)$$

Simultaneously substituting the boundary condition Equations (20) and (21) into Equation (30):

(31) $${\left[d_{ij}\right]}_{5\times 5}{\left[\begin{array}{ccccc} A & B & A_{1} & B_{1} & A_{2} \end{array}\right]}^{T}={\left[\begin{array}{ccccc} 0 & 0 & 0 & 0 & 0 \end{array}\right]}^{T}$$

In order to make the Equation (31) have a non-zero solution, the coefficient determinant must be zero:

(32) $$\left|d_{ij}\right|=0\;(i,\;j=1,2,3,4,5)$$

Equation (32) is the dispersion equation of the UGWs in the tube filled with liquid.

#### 2.1.3. For Concrete Filled Tubular Structures

As shown in Figure 1, when the internal material is concrete, its outer boundary stress is zero, and it is assumed that the coupling effect between the steel pipe and concrete is rigid, and the internal boundary condition is described as [26]:

(33) $$\begin{array}{ll} R_{1}=a & \sigma_{rr}=\sigma_{rz}=0\\ R_{2}=b & \left\{ \begin{array}{ll} \sigma_{rr1}=\sigma_{rr2} & \sigma_{rz1}=\sigma_{rz2}\\ u_{r1}=u_{r2} & u_{z1}=u_{z2} \end{array}\right. \end{array}$$

According to elastic mechanics theory, when guided waves are propagating in a structure, the displacement of the particles must satisfy the Navier wave equations of motion [14,25], as shown in Equation (1).

When there is no external force, the displacement vector *U* can be decomposed into an expression of an expansion scalar potential function φ and an equal volume vector potential function *H* according to the Helmholtz decomposition law, as shown in Equation (2), in which, ∇⋅H=0. For the Equation (1), the potential function φ and *H* should be satisfied Equations (3) and (4).

For the propagation of guided waves in a pipe structure, Gazis [14] first studied and gave the exact solution of this boundary condition:

(34) $$\left\{ \begin{array}{l} \varphi =f(r)\cos n\theta \cos(\omega t+kz)\\ \mu_{r}=g_{r}(r)\sin n\theta \sin(\omega t+kz)\\ \mu_{\theta }=g_{\theta }(r)\cos n\theta \sin(\omega t+kz)\\ \mu_{z}=g_{z}(r)\sin n\theta \cos(\omega t+kz) \end{array}\right.$$

in which, ω is circular frequency; *k* is wave number; *n* is circumferential order of guided waves (0, 1, 2, 3,…); $\mu_{r}$,$\mu_{\theta }$ and $\mu_{z}$ are radial component, circumferential component and axial component in displacement field, respectively. $g_{r}(r)$, $g_{\theta }(r)$ and $g_{z}(r)$ are displacement amplitudes of radial direction, circumferential direction and axial direction, respectively. 

Liu [26] introduced the differential operator and obtained the general solution through the Bessel function. Combining Equation (2) and the vector algorithm in the cylindrical coordinate system, the displacement component of the guided wave in the r and z directions in the longitudinal mode is obtained as:

(35) $$\left\{ \begin{array}{l} u_{r}=\frac{\partial \varphi }{\partial r}-\frac{\partial H_{\theta }}{\partial z}=\left(f^{\prime }-ikg_{\theta }\right)\cos(\omega t+kz)\\ u_{z}=\frac{\partial \varphi }{\partial z}+\frac{1}{r}\frac{\partial \left(rH_{\theta }\right)}{\partial z}=\left(ikf+\frac{g_{\theta }}{r}+g_{\theta }^{\prime }\right)\sin(\omega t+kz) \end{array}\right..$$

Stress components are:

(36) $$\left\{ \begin{array}{l} \sigma_{rr}=\left[-\lambda \left(\alpha^{2}+k^{2}\right)f+2\mu \left(f^{\prime \prime }-ikg_{\theta }^{\prime }\right)\right]\cos(\omega t+kz)\\ \sigma_{rz}=\mu \left[2ikf^{\prime }+\left(k^{2}-\beta^{2}\right)g_{\theta }\right]\sin(\omega t+kz) \end{array}\right..$$

The Equations (35) and (36) are brought into the boundary conditions (33), producing a set of characteristic equations:

(37) $$\begin{array}{cc} \left[M_{ij}\right]\cdot \left[N\right]=0 & i,j=1,2,\cdots 6 \end{array}.$$

In Equation (37), $\left[N\right]={\left[A\;B{\;A}_{1}{\;B}_{1}{\;A}_{2}{\;A}_{3}\right]}^{T}$, $M_{ij}$ is coefficient matrix. In order to make Equation (37) have non-zero solution, the coefficient determinant must be zero:

(38) $$\left[M_{ij}\right]=0$$

Then Equation (38) is the dispersion equation of longitudinal modal guided waves in concrete filled steel tubular structures. Shown in Table 1 is the Bessel function selection principle.

### 2.2. The Solution of Dispersion Equations

The frequency dispersion equation of guided waves in tubular structure members is a transcendental equation, and it can only be solved by numerical calculation. The group velocity and phase velocity have the following relation in guided waves [27]:

(39) $$c_{g}=\frac{c_{p}^{2}}{c_{p}-fd\cdot \frac{dc_{p}}{d\left(fd\right)}}$$

From Equations (19), (32), (38) and (39), we can obtain the guided wave group velocity and phase velocity dispersion curves in a hollow steel tube, a tube filled with liquid, and a concrete filled steel tube (CFST), respectively. The excitation frequency of the guided wave is iteratively calculated at a certain step. The relevant material parameters of tubular structures are shown in Table 2. The relevant parameters are obtained according to the relevant Chinese specifications. The specific specification is the Concrete Structure Design Specification of China (GB50010-2010).

**Figure 2:**
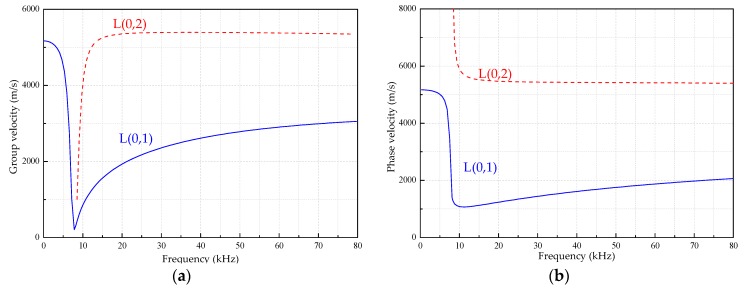
The frequency dispersion curves in the range of 0~80 kHz for a hollow steel tube. (**a**) The group velocities. (**b**) The phase velocities.

**Figure 3:**
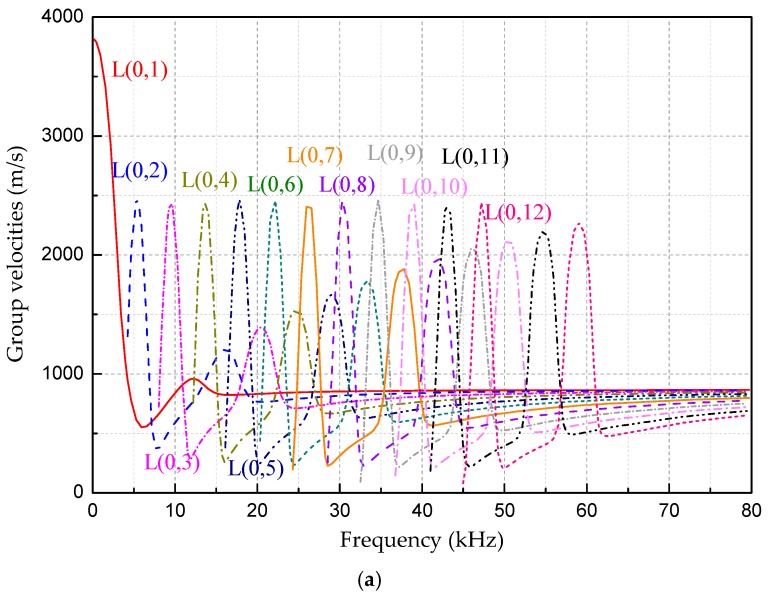

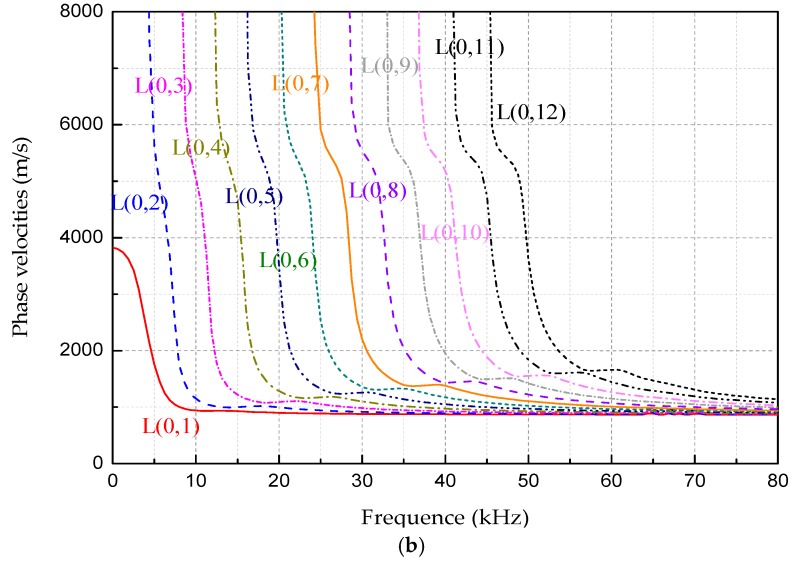
The frequency dispersion curves in the range of 0~80 kHz for a tube filled with liquid. (**a**) The group velocities. (**b**) The phase velocities.

**Figure 4:**
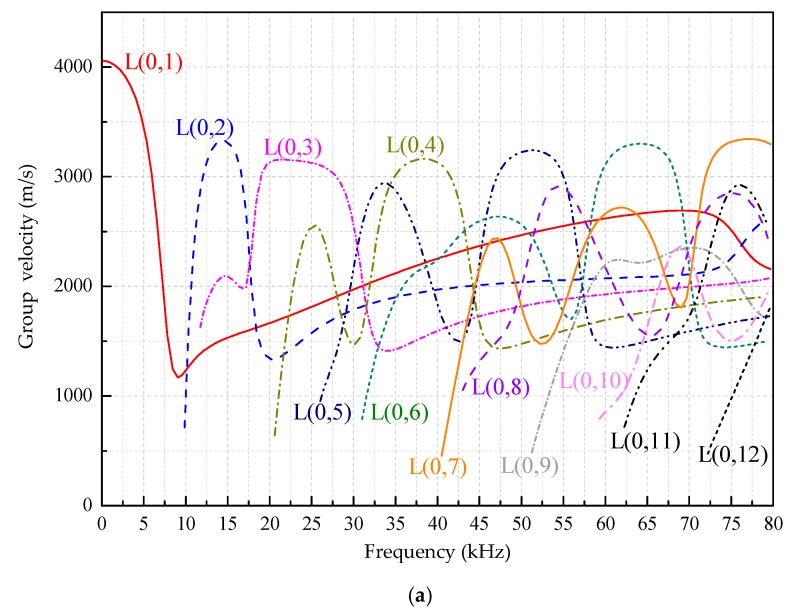

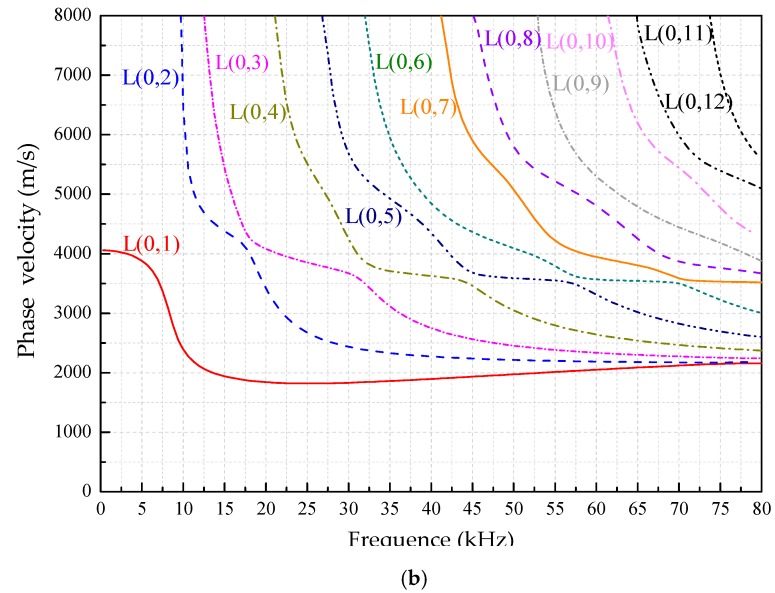
The frequency dispersion curves in the range of 0~80 kHz for a CFST column filled with concrete of C30. (**a**) The group velocities. (**b**) The phase velocities.

Figure 2, Figure 3 and Figure 4 are the frequency dispersion curves for tubular structures with different interfacial boundary conditions.

From Figure 2, Figure 3 and Figure 4, the following can be concluded:

(1) The ultrasonic guided wave propagation in tubular structures members obviously has the frequency dispersion and multi-modes characters, and phase velocities and group velocities vary with change of actuation frequencies.
(2) At a given frequency, two (or more than two) modes are generated at the same time except at a lower frequency range, but the group velocity (phase velocity) at each mode is different, and this situation is especially more obvious at higher frequency range than that at lower range.
(3) In addition to the L(0, 1) mode, there is a cutoff frequency for other modes of guided waves. That is, the guided wave with the mode at higher than cut-off frequency range can be propagated and the guided wave with the mode is rapidly decaying and not propagating below the cutoff frequency.

**Figure 5:**
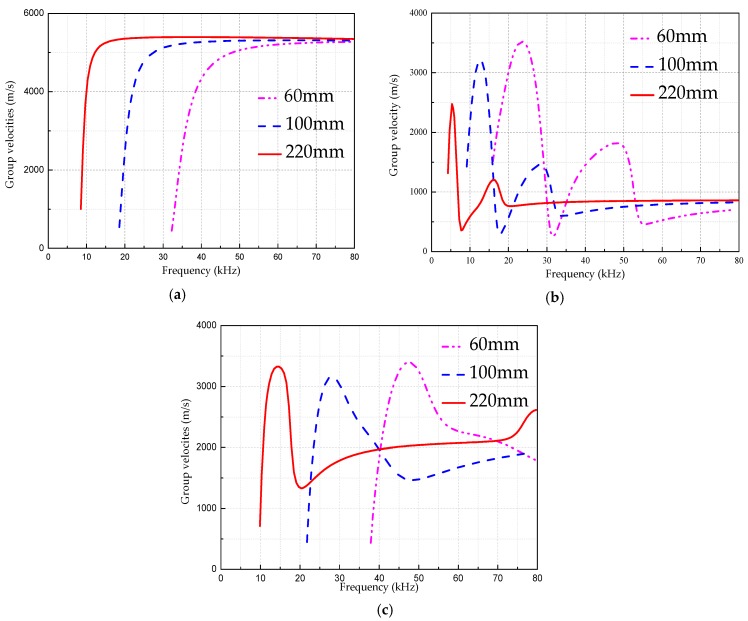
The comparison of the L(0, 2) mode frequency dispersion curves for various boundary conditions with different pipe diameters. (**a**) The hollow steel tube. (**b**) The tube filled with liquid. (**c**) The concrete filled steel tube.

In order to study the influencing factors of the dispersion curves, different parameters were changed under various boundary conditions, and the same modal curve was drawn for comparison. Since typical specifications in China are 14–720 mm in diameter for steel tubes, 60 mm for water pipes, and 220 mm for CTSTs which are commonly used in engineering. Therefore, without loss of generality, several tubes with diameters in this range are selected for the comparative study. Due to the L(0, 2) mode frequency dispersion curves are typically and widely applied in DDs and SHMs, the figure with L(0, 2) mode is used as an example to demonstrate the dispersion characteristics. Figure 5 is a comparison of the L(0, 2) mode curves for various boundary conditions at different diameters. Figure 5 shows that the cut-off frequency of the curves trend to move to high frequency with the decrease of the tubular structure diameters.

From Figure 5 we can find that for the same wall thickness, when the diameter increases, the dispersion curve trends to gradually move to the low frequency region with the obvious shift of cut-off frequency and the weak change of peak value in the curve.

**Figure 6:**
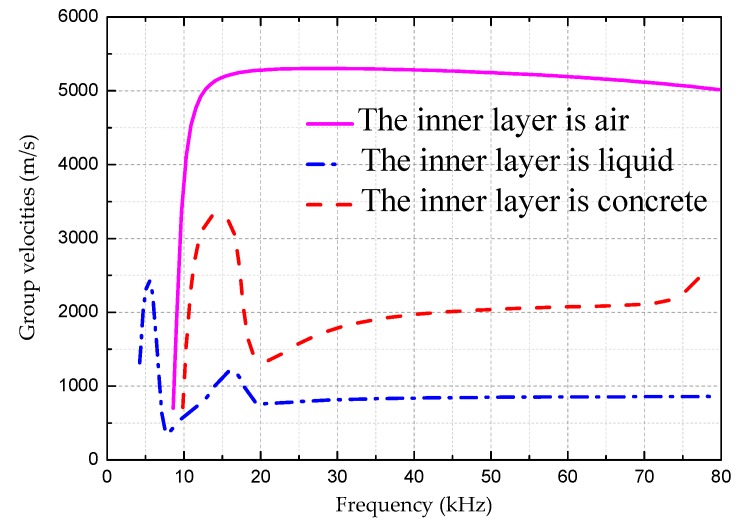
The comparison of L(0, 2) mode curves for various boundary conditions for the same pipe diameter of 220 mm.

Figure 6 is the L(0, 2) mode comparison curve for various boundary conditions for the same pipe diameter. For the pipe with air in it, the group velocity is the highest with a weak dispersive effect. For the pipe filled with liquid, the group velocity is lower than that filled with air but higher than that filled with concrete. For the pipe filled with concrete, the group velocity is the lowest one. For the pipe filled with liquid or concrete, at the low frequency range, the frequency dispersion effect is obvious which is difficult for DDs and SHMs, but it becomes much easier at the high frequency range due to the weak dispersion effects.

It can be seen from Figure 5 and Figure 6 that the UGWs propagation in different interfaces has dispersion and multi-mode characteristics, which are not only related to the product of frequency and thickness, but also the internal dielectric material parameters and interfacial boundary conditions.

## 3. Experimental Verification of Dispersion Curves

**Figure 7:**
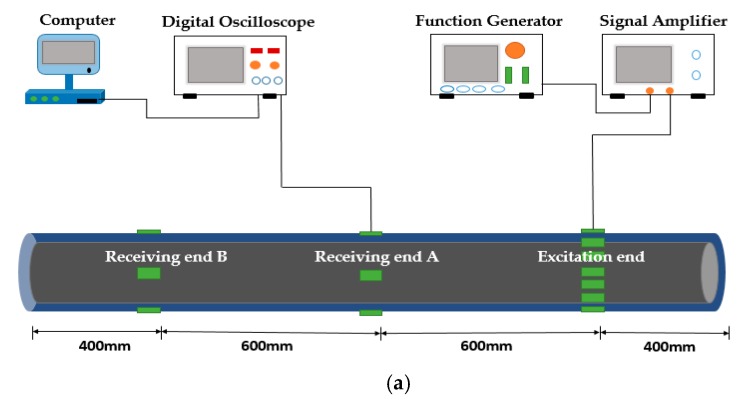

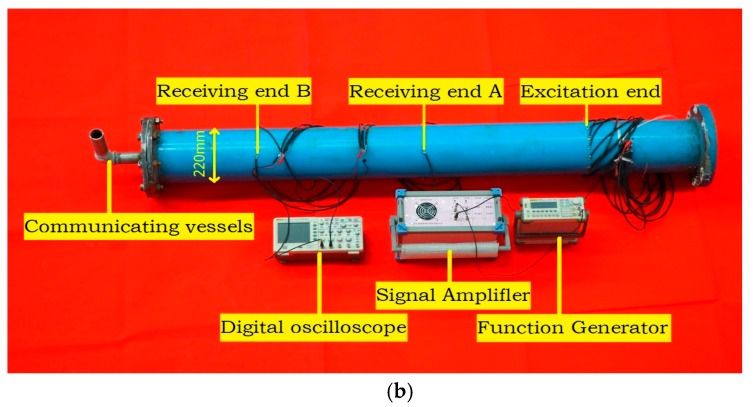
The schematics of PZT-based UGWs propagation experimental system. (**a**) The schematic diagram. (**b**) The experimental setup.

The objective of the experiment is to validate the accuracy for the dispersion curves of the tubular structures under different internal boundary conditions. Therefore, an experimental system that uses piezoceramics as transducers to excite and receive ultrasonic guided waves is designed. As shown in Figure 7, the tubular structure is 2 m long, the diameter is 220 mm, and the wall thickness is 6 mm, which is filled in air (hollow), water and concrete, respectively. The tested pipe is sealed at one end and a communicating vessel at the other end to ensure that the liquid in the tube is fully filled. The experimental setup includes a function generator for generating the guided wave with the given frequency, a signal amplifier for amplifying the signal voltage to meet the requirement of the experiment, and a digital oscilloscope for receiving and restoring the data. The tubular structure is used as the test specimen where piezoceramic (lead zirconate titanate, PZT) patches are pasted on the surface of it to be used as transducers. At the excitation end, a group of 16 PZT patches are used as actuators to generate a guided wave of L(0, 2) mode with the expected frequencies [25]. The selection of the L(0, 2) mode is because the UGW with the mode is not only typically and widely applied in DDs and SHMs, but also has the fast propagation velocity, weak frequency dispersion, and the minimized mode conversion and superposition effects at boundaries, which is much beneficial for the data processing of the received guided waves. The other group of PZT patches is used as sensors which are separately located at the positions A and B, 600 mm away from each other. The actuators are activated to generate the guided waves propagating along the tubular structures and being received simultaneously at two points of A and B. The arrival time difference of the signal packages can be extracted to calculate the group velocities according to the given distance by using the time of flight method.

**Figure 8:**
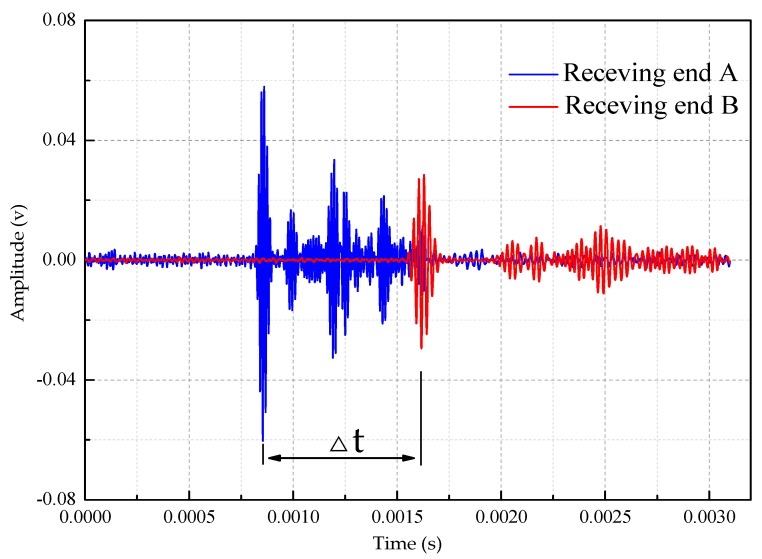
Signal received at A and B points when water is filled in the tubular.

As shown in Figure 8, a five peak impulse signal modulated by Hanning window function is applied in the tubular structure which is filled in water and taken as an example to show how to calculate the signal arrival time difference, and the wave arrival time corresponding to the peak of the first arrival wave package is used to calculate the time arrival difference. The center frequency of the signal is 70 kHz and the excitation amplitude is ±10 V. 

It can be seen from Figure 8, the time difference between the head wave of the ultrasonic guided wave reaching the two points A and B is:

$$\Delta t=t_{a}-t_{b}=0.0007345\;s$$

It is known that the distance between two points of A and B is 600 mm, then the wave velocity can be calculated as:

$$V=\frac{S}{\Delta t}=\frac{0.6}{0.0007345}=816.88\;m/s$$

The same method is used to measure the signal time difference for the three kinds of interfacial boundary conditions. Table 3, Table 4 and Table 5 is the measured value of the guided wave velocity at different frequencies under different boundary conditions, respectively. 

**Figure 9:**
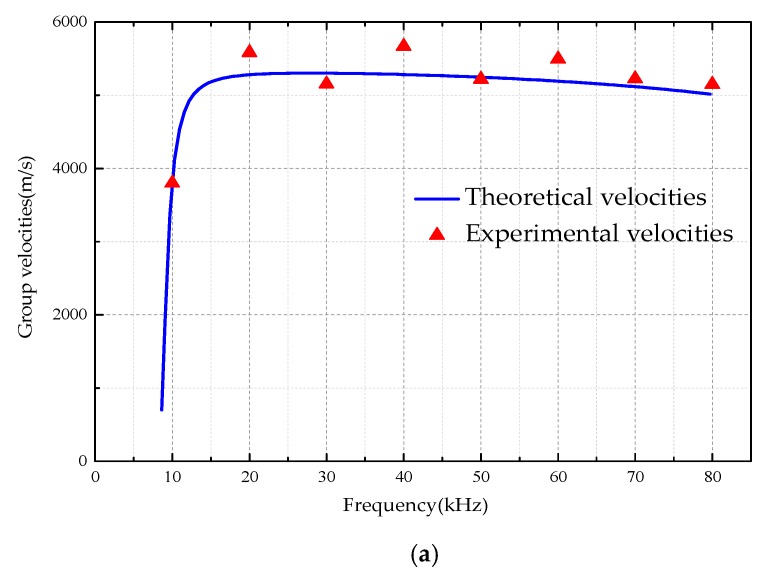

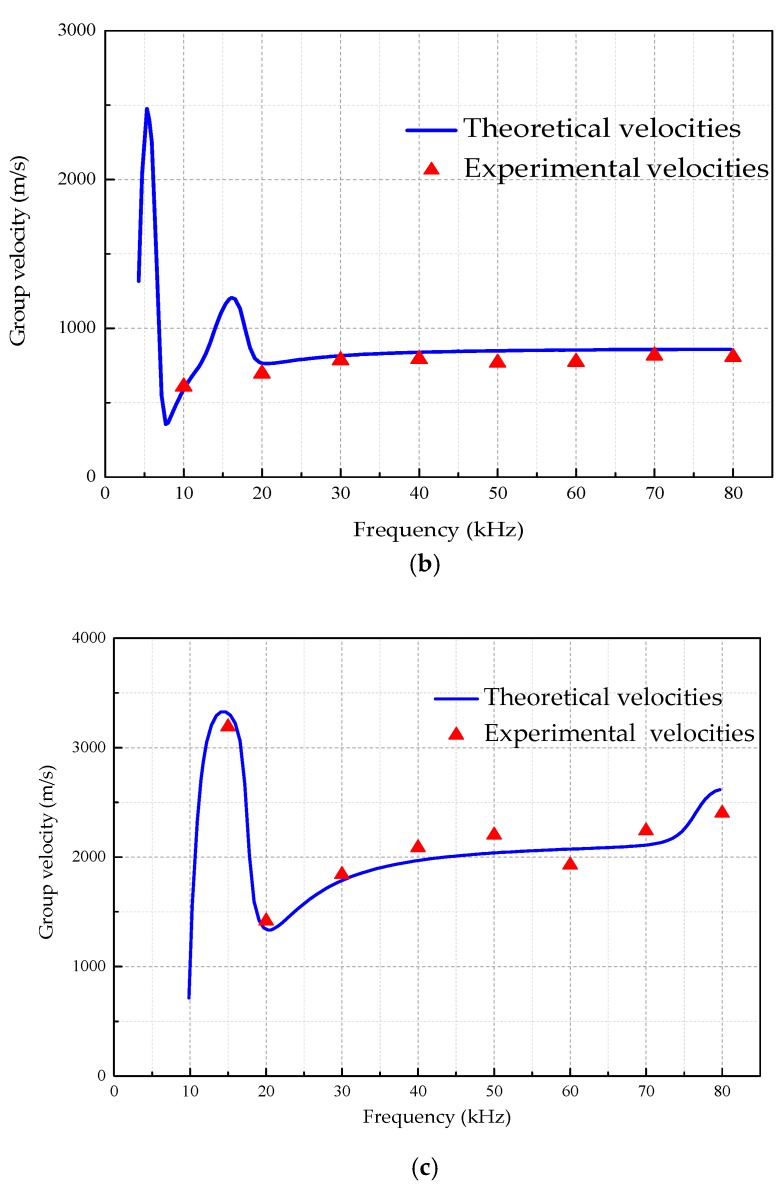
A comparison of guided wave velocity under different boundary conditions. (**a**) The tubular structure is filled in air (hollow). (**b**) The tubular structure is filled in water. (**c**) The tubular structure is filled in concrete.

Figure 9 is a comparison of guided wave velocity under different boundary conditions.

From Table 3, Table 4 and Table 5 as well as Figure 9, due to the accuracy of the test setup and the interference of the on-site environment, there is a certain error between the measured value and the theoretical one, but it can be seen that the maximum error between theoretical results is very close. This validates the correctness and effectiveness of the dispersion curves of the UGWs propagating in the tubular structures under different boundary conditions.

## 4. Damage Identification Experiment

**Figure 10:**
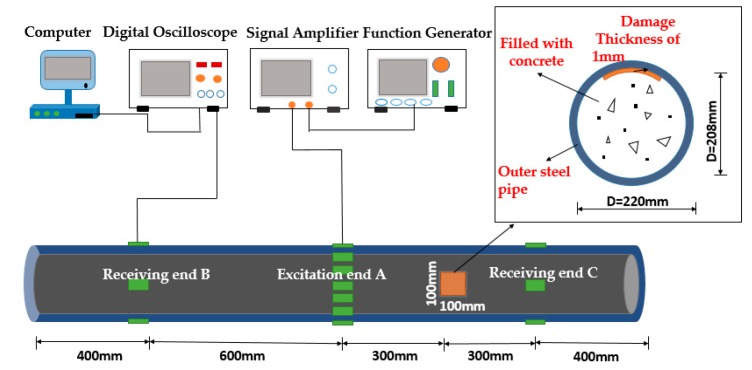
The schematic of damage identification experimental system.

In order to validate the efficiency of theoretical analysis results and better use the dispersion curves to detect the interface damages, an experimental system is designed as shown in Figure 10. The tested object is a CFST column with artificial interfacial damage. The CFST column has a length of 2 m, a diameter of 220 mm and a wall thickness of 6 mm, and the concrete strength is C30 according to the corresponding design code of China. A thin film of 100 mm × 100 mm × 1 mm is artificially arranged inside the pipe wall before casting concrete to simulate interfacial debonding damage between concrete and steel tubular wall. The thickness of the thin film is 1 mm to simulate slight damage in the actual situation. The width (length) of the sheet of 100 mm is selected because of one more wavelength of the actuated UGW which is more conducive to damage detection. PZTs are arranged and symmetrically pasted on the surface of the CFST column at A, B and C locations, respectively. Point A is the excitation location, and point B and C are the reception points. It is assumed that the AB segment is in a healthy state, and the BC segment is in a damaged state. The two segments will be used to compare for identifying the damage by UGW-based method. The experimental setup is as the same as the above-mentioned one.

**Figure 11:**
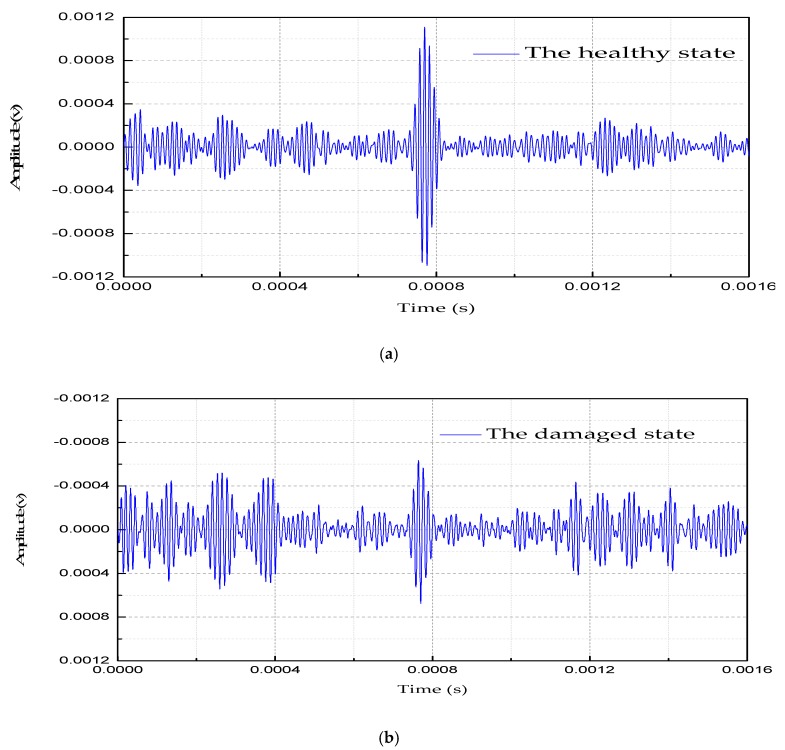
The filtered signal curve in time-domain measured at the central frequency of 60 kHz. (**a**) The healthy state. (**b**) The damaged state.

As shown in Figure 11, a five peak impulse signal modulated by Hanning window function is applied in the CFST column. The signal with the center frequency of 60 kHz and the excitation amplitude of ± 10 V is activated, the L(0, 2) mode guided wave is simultaneously propagating along two direction of the CFST column, and the signals are received by PZT sensors located at the points A and B, respectively. 

From Figure 11, we can see that the signal amplitude in the healthy state is obviously greater than that in a damaged state. This is because the existence of the interfacial damage weakens the propagation of the UGWs and causes the sensor signal energy to decrease. The signal energy-based method is used to set up the damage identification variables and index to experimentally evaluate the damage level. 

The amplitude of a sensor signal is an ideal parameter for damage identifications by using wave-based method. In general, damages may attenuate the amplitude of the sensor signal, and the amplitude attenuation degree may increase with development of the damages. The amplitude of the sensor signal is one of the external manifestations of the UGW energy. Therefore, the energy of the sensor signal can be used as a characteristic parameter to qualitatively identify structural damages. [24,28]. The sensor signal is a group of discrete values and the signal energy can be calculated by Equation (40), and the signal energy is applied as the damage identification variable:

(40) $$E={\sum_{n=-\infty }^{\infty }\left|x(n)\right|}^{2}$$

in which, *x*(*n*) is the signal corresponding to the discrete sequence; *n* is the sampling point.

The guided wave signal will undergo the energy attenuation during the propagation in component, and the relative percentage of the energy of the received signal to that of the excitation signal is defined as the attenuation index. As shown in the Equation (41):

(41) $$\alpha =\frac{E}{E_{m}}$$

where α is the attenuation index; *E* is the sensor energy which can be calculated by Equation (40); *E*_m_ is the actuation signal energy, which comes from the oscilloscope reading by directly connecting the actuation electrical wire to the oscilloscope.

**Figure 12:**
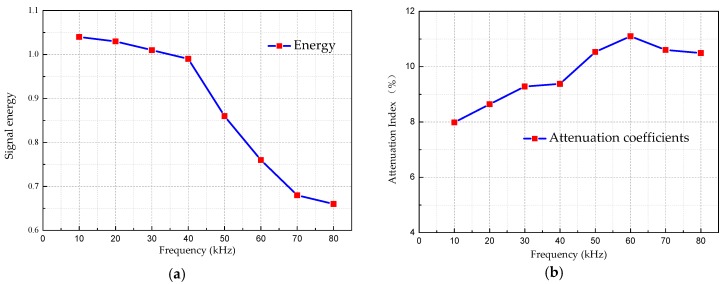
Changes of energy and energy ratio at different frequencies. (**a**) The relationship between energy and frequency. (**b**) The relationship between attenuation coefficients and actuation signal frequencies.

When the performance of the structural material is unchangeable, the attenuation of the signal energy is mainly affected by the excitation frequency. As shown in Table 6 is the relationship between the excitation frequency and the received energy and attenuation coefficient. Figure 12 is the relationship curve between the attenuation coefficient and the excitation frequencies.

As can be seen from Figure 12a, as the frequency increases, the signal energy attenuation is more obvious. The attenuation coefficient increases with the increase of frequency at low frequency range, and becomes nearly flat after reaching 60 kHz, as shown in Figure 12b.

**Figure 13:**
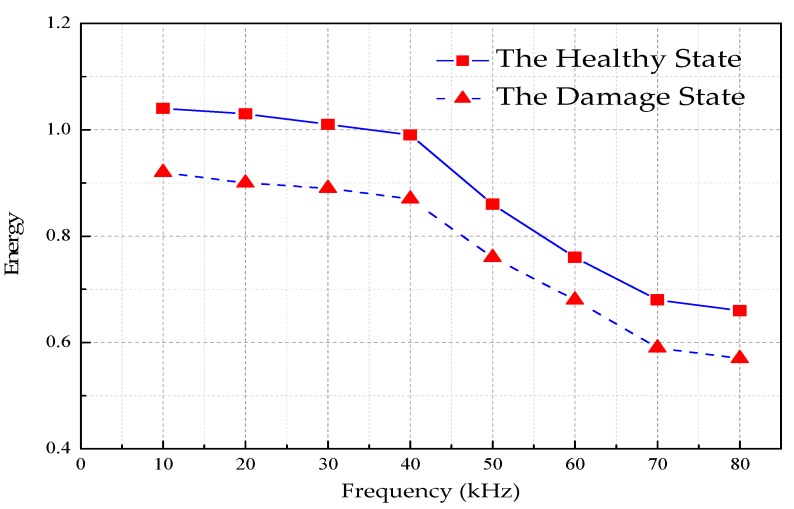
The comparison curve of received signal energy under healthy state and damage state.

The UGWs with the same frequency is actuated, and the guided wave is propagating along the healthy CFST segment and the damaged CFST segment, and all other parameters are the same except the damage. The received signal energy values are shown in Table 7. The comparison curve of received signal energy under healthy state and damage state is shown in Figure 13.

It can be seen from the Figure 13 that after the UGWs with the same frequency propagate for the same distance in the CFST, but the energy attenuation is different between the healthy state and the damaged one. In the damaged state, the energy attenuation is more obvious than that in the healthy one. The percentage of the energy value in the damaged state and the healthy state is redefined as the damage variable, as shown in Equation (42):

(42) $$H=\frac{E_{r}}{E_{h}}$$

where *E*_r_ is the sensor signal energy ratio for the damaged state; *E*_h_ is the sensor signal energy ratio for the healthy state. For the healthy state, *E*_r_ = *E*_h_ , and *H* = 1. Therefore, the corresponding damage index can be defined as Equation (43):

(43) $$D=1-H=\frac{E_{h}-E_{r}}{E_{h}}\times \%$$

As shown in Table 8 is the damage index value at each frequency, and Figure 13 is the relationship curve between the damage index and excitation frequencies.

**Figure 14:**
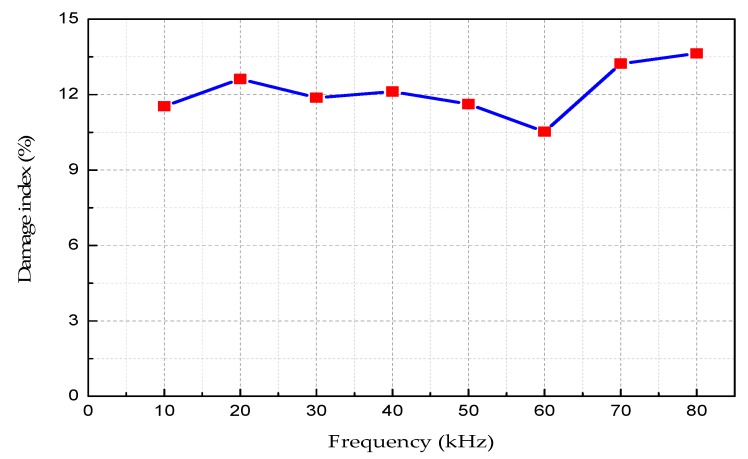
The damage index curve vs. the excitation frequencies.

As can be seen from Figure 14:

(1) Under the same damage conditions, the damage index does not change significantly with different excitation frequencies.
(2) The damage index value is in the range of 0 and 1. For the healthy state, *D* = 0; for the damaged state, 0 < *D* < 1. When the damage index *D* tends to zero, the CFST structure is prone to be healthy; when the damage index *D* tends to increase, the CFST might be damaged and the greater value of *D* means the more serious damages.

## 5. Conclusions

In this paper, the UGW propagation characteristics in different interfaces are classified and discussed, and the frequency dispersion curves for both group and phase velocities of UGWs are drawn. To validate the efficiency of theoretical and numerical results, three kinds of model tubular structures experiments filled in air (hollow), water and concrete, respectively, are performed using lead zirconate titanate (PZT) transducer-based UGWs. Obviously, the maximum error between theoretical results and experimental ones is very small, validating the correctness and effectiveness of the dispersion curves of the UGWs propagating in the tubular structures under different boundary conditions:

(1) From the comparison curves we can see that the propagation of UGWs in different interfaces has typical dispersion and multi-modes characteristics, which are not only related to the product of frequency and thickness, but also to the internal dielectric material parameters and interfacial boundary conditions.
(2) From the comparison among tubular structures filled with air, liquid and concrete, the changes of the interfacial boundaries (or connections) result in complicated dispersion characteristics for the propagation of UGWs, which increase the difficulty for DDs and SHM using UGW-based methods.
(3) After the UGWs propagate through the damage, the energy attenuation in the damaged tubular structure is more obvious than it is in a healthy one. The percentage of the energy value in the damaged state and the healthy state is defined as the damage index *D*. For the healthy state, *D* = 0; for the damaged state, 0 <*D* < 1. When the damage index *D* tends to zero, the CFST structure is prone to be healthy; when the damage index *D* tends to increase, the CFST might be damaged and the greater value of *D* means the more serious damages.

## Figures and Tables

**Table 1:** Bessel function selection principle.

Section	Functions	
$c_{L}$ *<* cp	$Z_{n}\left(\alpha r\right)=J_{n}\left(\alpha r\right)W_{n}\left(\alpha r\right)=Y_{n}\left(\alpha r\right)$	$Z_{n}\left(\beta r\right)=J_{n}\left(\beta r\right)W_{n}\left(\beta r\right)=Y_{n}\left(\beta r\right)$
$c_{T}$ *<* cp *<* $c_{L}$	$Z_{n}\left(\alpha r\right)=I_{n}\left(\alpha r\right)W_{n}\left(\alpha r\right)=K_{n}\left(\alpha r\right)$	$Z_{n}\left(\beta r\right)=J_{n}\left(\beta r\right)W_{n}\left(\beta r\right)=Y_{n}\left(\beta r\right)$
cp *<* $c_{L}$	$Z_{n}\left(\alpha r\right)=I_{n}\left(\alpha r\right)W_{n}\left(\alpha r\right)=K_{n}\left(\alpha r\right)$	$Z_{n}\left(\beta r\right)=I_{n}\left(\beta r\right)W_{n}\left(\beta r\right)=K_{n}\left(\beta r\right)$

**Table 2:** The relevant material parameters of tubular structures.

Material	External Diameter (mm)	Internal Diameter (mm)	*E* (GPa)	ρ (kg/m^3^)	μ
Steel Tube	220	208	206	7850	0.3
Concrete	208	0	30	2400	0.2
Liquid	208	0	2.18	1000	0.5

**Table 3:** Theoretical and experimental values in the tubular which is filled in air.

Frequencies (kHz)	10	20	30	40	50	60	70	80
**Theoretical velocities (m/s)**	3741	5280	5301	5283	5246	5192	5117	5015
**Experimental velocities (m/s)**	3799	5582	5154	5669	5219	5495	5224	5146
**Error (%)**	1.55	5.72	2.77	7.31	0.51	5.84	2.09	2.61

**Table 4:** Theoretical and experimental values in the tubular which is filled in water.

Frequencies (kHz)	10	20	30	40	50	60	70	80
**Theoretical velocities (m/s)**	588	768	817	838	849	854	858	859
**Experimental velocities (m/s)**	608	695	785	792	768	773	817	806
**Error (%)**	3.40	9.51	3.92	5.49	9.54	9.48	4.78	6.17

**Table 5:** Theoretical and experimental values in the tubular which is filled in concrete.

Frequencies (kHz)	15	20	30	40	50	60	70	80
**Theoretical velocities (m/s)**	3327	1343	1786	1968	2038	2074	2110	2617
**Experimental velocities (m/s)**	3189	1416	1841	2084	2200	1927	2239	2399
**Error (%)**	4.15	5.44	3.08	5.89	7.95	7.09	6.11	8.33

**Table 6:** Energy value and attenuation coefficient at each frequency level.

Frequencies (kHz)	10	20	30	40	50	60	70	80
**Energy**	1.04	1.03	1.01	0.99	0.86	0.76	0.68	0.66
**Attenuation index (%)**	7.98	8.64	9.28	9.37	10.5	11.1	10.6	10.4

**Table 7:** The received signal energy values.

Frequencies (kHz)	10	20	30	40	50	60	70	80
**Healthy State**	1.04	1.03	1.01	0.99	0.86	0.76	0.68	0.66
**Damaged State**	0.92	0.9	0.89	0.87	0.76	0.68	0.59	0.57

**Table 8:** The damage index value at each frequency.

Frequency (kHz)	10	20	30	40	50	60	70	80
**Damage index (%)**	11.54	12.62	11.88	12.12	11.63	10.53	13.24	13.64
